# Topical Triamcinolone Acetonide-Loaded Liposome Formulation Used as an Adjuvant to Intravitreal Ranibizumab Therapy for Neovascular Age-Related Macular Degeneration

**DOI:** 10.3390/pharmaceutics13091491

**Published:** 2021-09-17

**Authors:** Jose Navarro-Partida, Juan Carlos Altamirano-Vallejo, Luis Abraham Aceves Franco, Jesús Gonzalez-Cortes, Sergio Hernandez-Da Mota, Jose Gerardo García-Aguirre, Carlos David Azuara-Galindo, Carlos Rodrigo Castro-Castaneda, Juan Armendariz-Borunda, Arturo Santos

**Affiliations:** 1 Tecnologico de Monterrey, Escuela de Medicina y Ciencias de la Salud, Monterrey 64849, Mexico; josenavarro@tec.mx (J.N.-P.); jcaltamirano@e-retina.com (J.C.A.-V.); A00832512@tec.mx (L.A.A.F.); jose.garcia.aguirre@tec.mx (J.G.G.-A.); crodrigocastro@gmail.com (C.R.C.-C.); armdbo@gmail.com (J.A.-B.); 2 Centro de Retina Medica y Quirúrgica, S.C., Hospital Puerta de Hierro, Zapopan 45116, Mexico; 3 Facultad de Medicina y Hospital Universitario “Dr. José Eleuterio González”, Universidad Autónoma de Nuevo León, Monterrey 64460, Mexico; jesus.gonzalezcrt@uanl.edu.mx; 4 Clinica David, Unidad Oftalmologia, Servicio de Retina, Morelia 58280, Mexico; tolodamota@yahoo.com.mx; 5 Asociacion para Evitar la Ceguera en Mexico I.A.P., Ciudad de Mexico 04030, Mexico; 6 ISSSTE Clinica Hospital Constitucion Monterrey, Monterrey 64530, Mexico; cazuaramd@smq.com.mx; 7 Department of Molecular Biology and Genomics, Institute for Molecular Biology and Gene Therapy, University of Guadalajara, Sierra Mojada 950, Colonia Independencia, Edificio Q. Tercer Piso, Guadalajara 44340, Mexico

**Keywords:** triamcinolone acetonide, liposomes, neovascular age-related macular degeneration, wet macular degeneration, adjuvant therapy, ranibizumab

## Abstract

Novel strategies have been developed to reduce or avoid intravitreal injections (IVTs) of the antiangiogenic (ranibizumab (RBZ)) and anti-inflammatory (triamcinolone acetonide (TA)) agents used to treat vitreoretinal diseases. One of the strategies includes liposomes. This study evaluated the safety and efficacy of a topical triamcinolone-loaded liposome formulation (TALF) as an adjuvant to intravitreal RBZ therapy in treatment- naïve patients with neovascular age-related macular degeneration (nAMD). Subjects were randomly assigned to the RBZ-TALF or the RBZ-pro re nata (RBZ-PRN) groups. Patients from the RBZ-TALF group were instructed to apply TALF for 12 months after a single dose of RBZ. Patients from the RBZ-PRN group received three monthly RBZ-IVTs. Retreatment with RBZ was considered in the case of nAMD reactivation. Regarding safety, non-ocular abnormalities were observed during TALF therapy. Concerning efficacy, non-significant differences were identified in terms of visual acuity or central foveal thickness when the RBZ-PRN and RBZ-TALF groups were compared. It is worth noting that the average number of RBZ injections was significantly lower in the RBZ-TALF group (2.5 ± 1.4 vs. 6.1 ± 1.3 IVTs; *p* = 0.0004). Therefore, TALF used as an adjuvant to RBZ reduces the need for RBZ-IVT retreatment with optimal visual and anatomic results.

## 1. Introduction

Age-related macular degeneration (AMD) has been targeted as the main cause of irreversible blindness in the population 50 years of age or older in developed countries [1,2,3,4]. Worldwide, around 17 million people live with this condition [3,5]. AMD can be classified into two categories: Neovascular (wet) and non-neovascular (dry). Although the neovascular form only represents between 10% and 20% of all cases of AMD, 80–90% of severe visual loss cases are explained by this form. Vision impairment in neovascular AMD (nAMD) follows the development of choroidal neovascularization in the macula. These vascular changes increase the fluid leakage with the consequent thickening and late-stage scarring of the central retina, leading to significant central vision loss [6].

Vascular endothelial growth factor (VEGF) has been demonstrated to be the most important angiogenic factor responsible for choroidal neovascularization (CNV) in nAMD. However, several lines of evidence implicate the immune system and different inflammatory pathways in nAMD pathogenesis. Chronic inflammatory cells such as fibroblasts, macrophages, and circulating progenitor/stem cells have been reported in surgically excised subfoveal neovascular membranes, as well as the accumulation of collagen, basal laminar deposits, and fibrin, which comprise the extracellular components [7,8,9,10,11,12]. It has also been described that IL-1-β and TNF-α secreted by macrophages may promote choroidal neovascularization [13]. Therefore, CNV is a process that involves inflammation and neovascularization. Owing to the participation of angiogenic mechanisms in the nAMD pathogenesis, molecules targeting angiogenesis (anti-VEGF agents), such as ranibizumab (RBZ), aflibercept, and the off-label bevacizumab, have been intravitreally used for over a decade to treat patients with nAMD, slowing disease progression and preserving and improving vision [14,15,16,17]. Intravitreal anti-VEGF therapy is currently the standard of care in patients with nAMD [18]. It is worthy of note that the anti-VEGF agents used in clinical practice, such as RBZ, bevacizumab, and aflibercept, are considerably different in terms of molecular interactions when they bind with VEGF [19]; therefore, characterization of such features can improve the design of novel biological drugs that are potentially useful in clinical practice.

Even though the benefits of intravitreal injections (IVTs) of anti-VEGF drugs in nAMD have been well documented, this procedure is linked to potential complications resulting from the route of administration, such as endophthalmitis, retinal detachment, and traumatic cataract [19,20]. Moreover, large boluses of anti-VEGF agents in long-term treatment have been related to macular atrophy [21,22].

In recent years, novel therapeutic strategies to reduce or avoid the use of intravitreal injections have been developed. These strategies include the improvement of treatment interval [23], sustained drug delivery systems [24,25], implantable drug delivery systems [26,27,28], and novel drug carrier systems such as microemulsions, nanosuspensions, nanoparticles, and liposomes [29,30,31,32]. One of these promising approaches is that of liposome-based nanocarriers. Liposomes are biocompatible vesicles that share structural characteristics, including a phospholipid bilayer, with the cell membrane and carry both hydrophilic and lipophilic drugs within their minute spheroid shape [33,34].

Recently, a triamcinolone acetonide-loaded liposome formulation (TALF) was designed to be topically used to release triamcinolone acetonide (TA), a well-known synthetic corticosteroid, into the vitreous cavity and reach the retinal tissue [35,36]. As previously described [36], its pharmacokinetic and physicochemical properties, including an acidic pH, high osmolarity, and a viscosity of 70 cP, as well as a compartmental diffusion, have shown to be important characteristics in clinical outcomes. The safety and tolerability of this novel liposomal formulation were successfully tested in rabbits and healthy volunteers [36,37], while its therapeutic activity was verified in patients with refractory pseudophakic cystoid macular edema, diabetic macular edema, and macular edema secondary to branch retinal vein occlusion [37,38,39]. TALF was demonstrated to enable the continuous, controlled, and effective release of triamcinolone acetonide into the posterior segment tissues and was also well-tolerated without eliciting adverse events such as ocular hypertension or cataract progression [38,39].

Since inflammatory and angiogenic pathways contribute to the development of neovascularization by increasing vascular permeability in the wet form of AMD, a therapy that suppresses both pathways has been postulated to produce sustainable effects and may have advantages compared to mono-therapeutic approaches [39,40]. Combined therapies with anti-VEGF agents and TA have shown positive effects in the treatment of nAMD and have become increasingly common in the past few years [41,42,43,44]. Due to the highly complex process represented by the pathogenesis of CNV, where both angiogenesis and inflammation play an important role, it is hypothesized that using topical ophthalmic TALF as an adjuvant to the intravitreal injection of RBZ could enhance the efficacy of the intravitreal anti-VEGF agent and minimize unwanted collateral effects by reducing the doses and the number of intravitreal injections already used in the monotherapy with RBZ. Therefore, in this clinical assay, we explored and reported the feasibility and safety of using topical TALF as an adjuvant to intravitreal anti-VEGF therapy with ranibizumab for nAMD.

## 2. Methods

### 2.1. Study Design

To evaluate the feasibility and safety of topical TALF used as an adjuvant to intravitreal anti-VEGF therapy with RBZ for nAMD, a single-center Phase II clinical trial was conducted in patients with a diagnosis of nAMD at a private-based, ISO 9001:20015 Certified research unit in ophthalmology in Guadalajara, Mexico (Centro de Retina Medica y Quirurgica, SC). An external review board’s approval authorization was obtained before the enrollment of patients (IJICSA Committee; ID: CRMQ-2015-05-T-02; approved on 13 December 2016; COFEPRIS 173300410A0035/2017). The study observed the tenets of the Declaration of Helsinki, and also adhered to the International Conference on Harmonization guidelines on Good Clinical Practices and all other applicable local regulatory requirements and laws. Before enrollment, written informed consent was obtained from all subjects after a full explanation of the nature and potential adverse events (AEs) of the study.

### 2.2. Patients

Subjects 55 years of age or older with active nAMD in one eye confirmed by fluorescein angiography (FA) were recruited. All angiographic subtypes of nAMD were enrolled. The study population comprised ranibizumab and any other anti-VEGF drug- or photodynamic therapy (PDT)-naïve patients. The other inclusion criteria were a best corrected visual acuity (BCVA) of 20/30 to 20/200 and the absence of other ocular diseases that could affect the BCVA. The key exclusion criteria were subretinal hemorrhage of the central area of the fovea (hemorrhage >50% of the total lesion area or one disc area in size), subfoveal fibrosis, or atrophy in the study eye. Patients with retinal vein/artery occlusion, diabetic retinopathy, bilateral nAMD, study eye refractive error >4 diopters of myopia, angioid streaks or precursors of CNV in either eye secondary to other etiologies, glaucoma, and any other subjacent retinal or optic nerve condition were also excluded from the study. Additional exclusion criteria included a medical history of myocardial infarction and/or stroke within six months before recruitment, any prior intravitreal anti-VEGF or corticosteroid injections, and any prior laser treatment and/or intravitreal steroid implants such as Ozurdex^®^.

### 2.3. Clinical Evaluation and Study Treatment

At baseline, all eligible participants underwent an ophthalmologic examination, including BCVA measurement with the early treatment diabetic retinopathy study (ETDRS) chart, fundus examination with a noncontact 90 diopter lens, slit-lamp examination, intraocular pressure (IOP) measurement using Goldmann applanation tonometry, FA (FF 450 Plus, Carl Zeiss Meditech, Inc., Dublin, CA, USA), and optical coherence tomography (OCT) (Cirrus 5000, Carl Zeiss Meditech, Inc., Dublin, CA, USA). The study eye of the subjects received a single IVT injection of 0.5 mg of ranibizumab at baseline. Then, the subjects were randomly assigned to one of two treatment groups: RBZ-TALF or RBZ-pro re nata (RBZ-PRN). Subjects from the RBZ-TALF group were instructed to instill in the surface of the study eye one drop of a liposomal formulation containing TA at 2 mg/mL (0.2%) four times a day for the entire follow-up (12 months). The 2 mg/mL (0.2%) concentration of TALF and the frequency of its instillation were established according to the data obtained from the pharmacokinetic study of tissue. First, the observed maximum concentration of TA was detected in intraocular tissues (vitreous and retina) after three drops. Second, the model proposed for the distribution and elimination of TA released by liposomes is compartmental, where vitreous humor behaves like a reservoir of TA that provides the drug to the retina [36]. Clinical evaluation and analysis of compliance to TALF therapy were scheduled as follows: Every week after the baseline visit for the first month and monthly until the end of the follow-up. Compliance of the subjects to the TA-LF therapy was evaluated through a patient care journal as follows: AD = (RA)100/IA, where AD means adherence, RA corresponds to the registered applications, and IA represents the indicated number of applications. A value of adherence <90% was considered as compliance failure and, in this case, the patient was excluded from the statistical analysis.

On the contrary, subjects from the RBZ-PRN group received two more IVTs of 0.5 mg of ranibizumab, one at the beginning of the second month and the other one at the beginning of the third month of the follow-up.

Retreatment with IVTs of 0.5 mg of RBZ (Lucentis, Novartis, S.A. de C.V., Mexico City, Mexico) was considered in the RBZ-TALF and RBZ-PRN groups, considering the PrONTO study criteria [45]. Additional reinjections were administered if certain changes were observed by the evaluating physician, including a loss in visual acuity of at least five letters compared to the highest BCVA with objective evidence of fluid in the macula by OCT, an increase of at least 100 µm regarding the CFT in OCT compared to the lowest CFT, a new appearance of macular hemorrhage, a new area of classic CNV, or the persistent presence of fluid on OCT at least one month after the previous injection. If retreatment (reinjection) was needed, the patient was instructed to return the next week and then for the next scheduled visit for follow-up.

### 2.4. Safety Assessment

The safety of TALF therapy as an adjuvant to intravitreal RBZ therapy was assessed through the collection and summary of ocular and non-ocular AEs at all study visits. It was recorded whether AEs were informed by the enrolled patients, determined by the study site personnel, or by any other means. Vital signs measurements were carried at baseline and monthly until the end of the follow-up period (12 months). Patients were removed from the study in the case of any evidence of low tolerability or any AEs related to the TALF topical therapy, such as ocular surface problems (i.e., corneal ulcers, opacities, epithelial defects, conjunctival, and/or episcleral injection) and anterior chamber inflammation (cell/flare). AEs were assigned standard codes for the event based upon the MedDRA Coding dictionary, version 18.1. The presence of AEs was evaluated each week throughout the first month and monthly for the rest of the follow-up period. To identify AEs, at each visit, evaluations with a slit-lamp of the anterior and posterior segments were performed and the IOP measurement was recorded.

### 2.5. Efficacy Assessment

The mean change from baseline in BCVA, retinal central foveal thickness (CFT), and the average number of ranibizumab retreatments during the follow-up period were considered as efficacy endpoints. The BCVA was evaluated with the ETDRS chart at 4 m, whereas the central foveal thickness (CFT) was evaluated by OCT (Cirrus 5000, Carl Zeiss Meditech, Inc., Dublin, CA, USA). Changes in FA (FF450 plus fundus camera Carl Zeiss Meditech, Inc., Dublin, CA, USA) were also evaluated. Certified technicians performed the BCVA measurement and the OCT and FA images acquisition at each visit.

### 2.6. TALF Preparation

OPKO Health, Inc. (Guadalajara, Jalisco, Mexico) provided the TALF. The preparation of TALF was carried out [46] as previously described. Briefly, the production of self-forming, thermodynamically stable TALFs (QuSomes^®^) was spontaneously accomplished upon adding polyethylene glycol glyceryl dimyristate (PEG-12) to an aqueous solution containing TA. The composition of TALF includes 2.0 mg of triamcinolone acetonide, 100 mg of PEG-12 glyceryl dimyristate, 0.8 mg of citric acid anhydrous, 14 mL of ethyl alcohol, 50 mg of kolliphor HS 15, 4.675 mg of sodium citrate dehydrate, 0.1 mg of benzalkonium chloride, and Q.S.1.0 m grade 2 purified water. The final TA concentration in the resultant dispersion was 2 mg/mL (0.2%). The preparation of TALF was performed in Good Manufacturing Practice (GMP) facilities and carried out as previously described. Sterility of the formulation was guaranteed by mixing the excipients of the formulation at 121 °C for 15 min and utilizing the filtration of buffers and water containing TA through membranes of a 0.22 μm pore size under aseptic conditions. The resulting formulation possessed a pH of 5.8, a viscosity of 70 cP, and an osmolarity of 334 mOsm/L. The dropper bottle containing TALF was preserved at room temperature.

### 2.7. Data Analysis and Statistical Methods

Data were analyzed using SPSS 22.0 software (IBM SPSS Statistics for Macintosh, version 22.0, IBM Corp, Armonk, NY, USA). Quantitative variables are described using the mean and standard deviation. Qualitative variables are described using frequencies and percentages. We performed a Mann–Whitney *U*-test for the analysis of BCVA, CFT, IOP, and the number of reinjections. For the analysis of gender and the study eye, a Fisher exact test was performed. Significance was defined as a *p*-value < 0.05.

## 3. Results

A total of 20 patients with a clinical diagnosis of nAMD were enrolled. Two patients of the RBZ-TALF group met the criteria for compliance failure and, therefore, were excluded from the statistical analysis. It is important to emphasize that compliance failure was secondary to concerns regarding the SARS-CoV-2 pandemic. Patients decided to suspend TALF therapy to avoid monthly clinical evaluations. The demographic and clinical characteristics of the patients and their study eyes are presented in Table 1.

**Figure 1:**
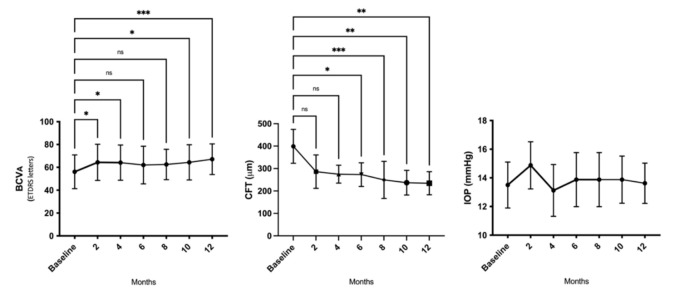
TALF improves BCVA and reduces CFT without IOP rising in patients with nAMD. TALF, triamcinolone acetonide-loaded liposome formulation; nAMD, neovascular age-related macular degeneration; BCVA, best corrected visual acuity; ETDRS, Early Treatment Diabetic Retinopathy Study; CFT, central foveal thickness; IOP, intraocular pressure; * *p* < 0.05, ** *p* < 0.01, and *** *p* < 0.001.

Regarding preliminary safety findings, non-serious AEs were related to TALF application during the follow-up period. Neither ocular surface abnormalities nor significant changes in IOP (Table 2 and Figure 1) or cataract progression in phakic eyes were observed. However, mild and transient ocular discomfort was reported. The recorded ocular symptoms were occasional mild dryness (25%), mild burning sensation (37.5%) during instillation, and momentary tearing after application (25%).

**Figure 2:**
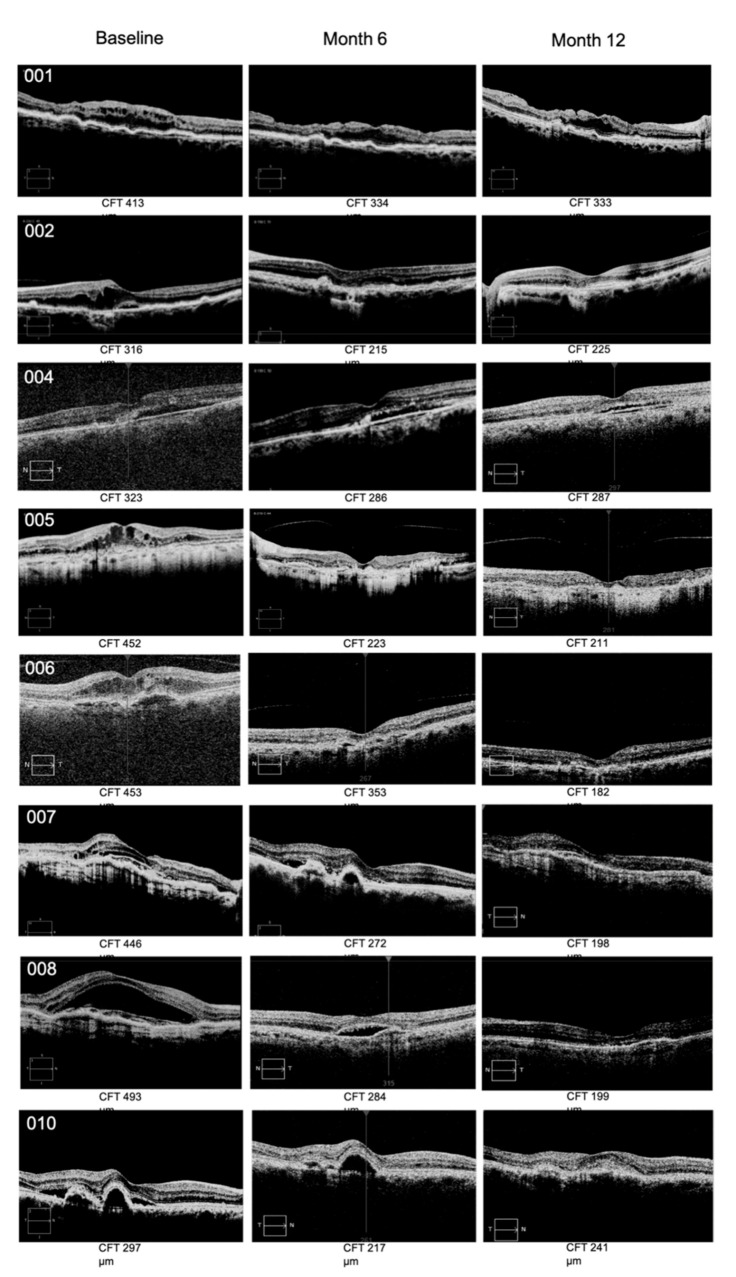
Representative images of CFT by OCT at baseline, month 6, and month 12 of the follow-up period for subjects included in the RBZ-TALF group. All patients showed a consistent and progressive reduction in CFT by month 12. CFT, central foveal thickness; OCT, optical coherence tomography; RBZ, ranibizumab; TALF, triamcinolone acetonide-loaded liposome formulation.

**Figure 3:**
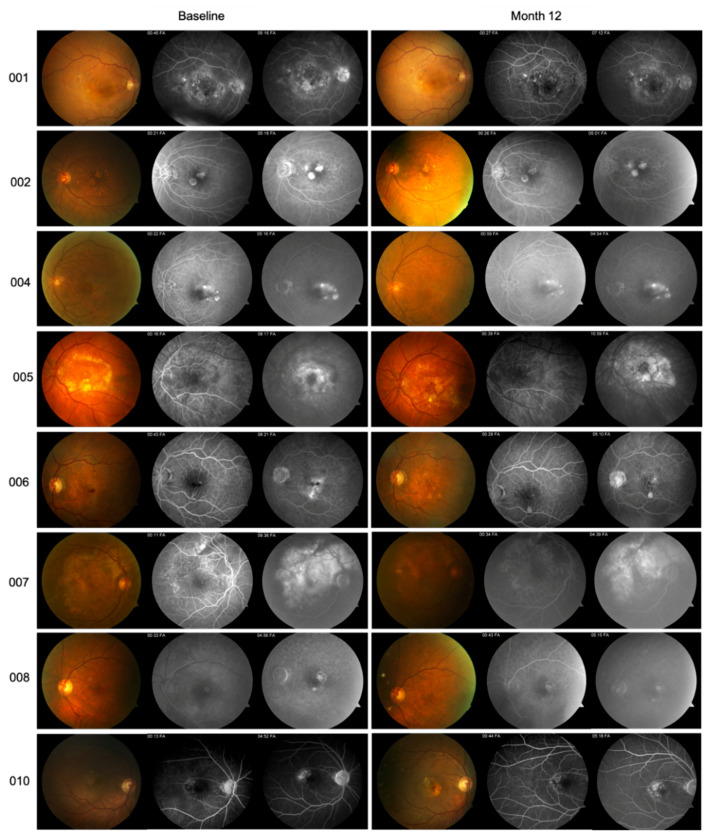
Representative images of fundus photography and fluorescein angiography at baseline and month 12 for subjects included in the RBZ-TALF group. All patients showed a decrease in active neovascularization activity.

Relative to efficacy outcomes, in the RBZ-TALF group, we observed a significant improvement in BCVA and CFT. BCVA improved from 56.12 ± 14.76 at baseline to 67.1 ± 13.4 letters (*p* < 0.002) at the end of the follow-up period (Table 2 and Figure 1). All patients improved in at least five letters, and four of them improved in more than 15 letters from baseline. Four out of the eight patients that completed the follow-up period had a BCVA of 70 letters or better at month 12 of the evaluation. CFT improved from 369.1 ± 80.5 μm at baseline to 271.3 ± 82.9 μm (*p* < 0.03) at month 12 (Table 2 and Figure 1). Representative OCT images are presented in Figure 2. Representative images of fundus and fluorescein angiography studies are presented in Figure 3.

Lastly, when the efficacy endpoints in the RBZ-TALF and RBZ-PRN groups were compared, non-significant differences were identified regarding BCVA or CFT (Table 3). Importantly, the average number of RBZ injections was lower in the RBZ-TALF group (Table 3). The mean number of injections in the RBZ-TALF group was 2.5 ± 1.4, whereas the average number of injections in the RBZ-PRN group was 6.1 ± 1.3 (*p* = 0.0004).

## 4. Discussion

Nowadays, intravitreal injections have become the route of preference for the intraocular delivery of anti-VEGF drugs [18]. Differing from other routes, IVTs have shown the advantage of delivering higher drug concentrations into the posterior segment, specifically into the vitreous and the retina, avoiding undesirable systemic effects. Additionally, IVTs surpass the blood–retinal barrier, which keeps most drugs out of the eye, including those administered by oral and systemic routes [47]. However, there are major challenges to be overcome. For instance, intravitreal drug administration creates a specific curve regarding its large peak concentration and rapid decay. The literature supports the fact that the half-life of anti-VEGFs, such as RBZ, is indeed very short (half-life of 2.6–4.0 days) [48], making it necessary to use a higher initial dose to exceed therapeutic levels to allow a longer 28-day treatment interval. Moreover, mathematical modeling demonstrates that the binding activity of 0.5 mg of RBZ is fivefold higher if given every 14 days instead of every 28 days [49]. Therefore, frequent intravitreal injections of ranibizumab are required to maintain the therapeutic effect and the visual outcomes [50,51]. Important clinical RBZ studies have suggested that patients should be treated with monthly injections [14,15,52,53]. However, intravitreal injections are not harmless. Each intravitreal injection presents with posterior administration risk and drug class-associated adverse events [54].

Furthermore, highly specialized human resources and special infrastructure are required for the administration of intravitreal injections, resulting in an expensive therapy option in developing countries [55]. Moreover, IVTs themselves cause discomfort to patients, turning them into a potential factor of poor adherence to anti-VEGF agents treatment [56]. Therefore, the frequency of intravitreal injection, which negatively impacts patients’ health and economy, is also a major challenge to overcome [55]. For these reasons, intravitreal injections might be a burden for physicians, the health system, and patients with poor compliance [55,56]. Diverse therapeutic approaches, including new drugs and medical devices, as well as modified therapeutic regimens such as treat and extend (T&E), have emerged to attend concerns about IVTs, even for reducing the burden of intravitreal therapy [57,58]. Although PRN (pro re nata) treatment protocols and treat and extend (T&E) regimens have been shown to elicit benefits [59,60,61,62,63], new and safer approaches are still needed.

This article reported the feasibility and safety findings of using TALF as an adjuvant to anti-VEGF intravitreal injections. Remarkably, a significant reduction in the number of ranibizumab intravitreal injections required to reach the inactivation of nAMD and maintain the visual outcomes that this anti-VEGF agent provides was observed. The average number of ranibizumab retreatments was 2.5 throughout 12 months, and all patients presented with a non-active nAMD, as evidenced by the FA study by the end of the follow-up period. This number of injections is surprisingly lower when compared to the reported number of injections in pivotal anti-VEGF studies. For example, one of the largest studies, the MARINA trial, a randomized and double-blind phase III sham-controlled trial designed to administer monthly injections of ranibizumab and posteriorly determine their efficacy and safety in the treatment of occult and minimally-classic neovascular age-related macular degeneration, reported an improvement in BCVA of 7.2 ETDRS letters with 13 intravitreal injections per patient throughout 12 months [64]. Meanwhile, the PrONTO trial, a non-randomized and prospective trial mainly designed to administer intravitreal RBZ every month for three months and to investigate its efficacy followed by a guided clinical and OCT appearances as-required dosing regimen, reported an improvement in BCVA of 9.3 ETDRS letters with 5.5 intravitreal injections per patient throughout 12 months [59]. In this TALF study, which included the application of topical liposomes containing triamcinolone as an adjuvant to intravitreal RBZ, we found an improvement in BCVA of 12.8 ETDRS letters with 2.5 intravitreal reinjections per patient throughout 12 months. Additionally, none of the patients enrolled in the TALF study presented with AEs. Therefore, TALF has the potential to reduce the treatment burden in nAMD by significantly reducing the number of retreatments. However, these preliminary findings should be confirmed in large cohort studies to rigorously determine the safety and efficacy of the TALF formulation.

Although the results are interesting, they were expected. TA is a synthetically modified glucocorticoid that is mainly used for its anti-inflammatory and immunomodulatory effects against several ocular diseases. Evidence indicates that TA decreases the secretion of VEGF into the human vitreous and the level of VEGF mRNA in cultured human retinal pigment epithelial cells with induced hypoxia or oxidation [65,66], and inhibits angiogenesis and the leakage of retinal blood vessels [13,67]. Moreover, TA has also shown a direct effect in the inflammatory process, embracing the inhibition of pro-inflammatory molecules such as tumor necrosis factor-alpha (TNF-α) and the pigment-derived growth factor (PEDF) [47,68,69,70]. Additionally, TA has been demonstrated to downregulate intercellular adhesion molecule 1 (ICAM-1) and reduce matrix metalloproteinase (MMP) expression on choroidal endothelial cells [71], influencing both angiogenesis and inflammation in nAMD.

However, intravitreal TA and the steroids related to it may elicit certain adverse effects, including increased intraocular pressure and cataract formation [72,73,74,75,76]. TALF is a topical formulation composed of liposomes that carry TA, which releases the drug into the vitreous and the retina in small but sustained amounts [35] and presumably extends the therapeutic effect of the anti-VEGF agents.

There are several studies in the literature evaluating the impacts of combined intravitreal injection of anti-VEGF agents and triamcinolone on AMD-affected eyes over time. For example, in a retrospective case series, Colucciello et al. treated 30 eyes of 27 patients afflicted with nAMD with a single combined intravitreal injection of bevacizumab (1.25 mg/0.05 mL) and triamcinolone (2 mg/0.05 mL). Their results revealed a statistically significant reduction in foveal thickness, as well as in subfoveal fluid volume (*p* < 0.01), eight weeks after the injection [77]. The same group conducted another interventional case-series study focused on 16 eyes of 16 patients presenting with nAMD who were previously treated with intravitreal mono-injections of bevacizumab (1.5 mg) without significant results regarding the reduction in macular exudation or improvement in visual acuity (VA) [78]. The participants of this investigation underwent a combined intravitreal injection containing bevacizumab (1.5 mg) and triamcinolone acetonide (about 20 mg). Significant improvement (*p* = 0.03) was observed concerning VA three months after the treatment. A similar prospective clinical trial by Tao and Jonas [43] revealed that intravitreal bevacizumab (1.5 mg) combined with intravitreal high-dose triamcinolone (20–25 mg) more effectively improves vision and reduces macular thickness in eyes with nAMD after unsuccessful intravitreal bevacizumab monotherapy. The findings of these studies demonstrate that triamcinolone acetonide, either at low (2 mg) or high (up to 25 mg) concentrations, can yield encouraging results in treating nAMD when administered intravitreally. As previously mentioned, intravitreal injection of TA is related to adverse effects such as increased intraocular pressure and cataract formation. These are the reasons why the use of TA or other glucocorticoids in nAMD is dismissed. We showed that TALF retains the therapeutic activity of TA for nAMD, but avoids the potential adverse events and complications of using TA IVTs. Remarkably, the use of TALF as an adjuvant to the intravitreal injection of RBZ for nAMD resulted in visual outcomes comparable to those attained with anti-VEGF PRN, but without non-ocular serious AEs, including IOP, rising. This result is coherent with those of previous studies, where the safety and efficiency of TALF have been documented for different vitreoretinal disorders [37,38,39].

On the contrary, a common concern about the therapeutic activity of topical TA for posterior segment disorders has to do with the reduced concentration of the drug achieved in the vitreous and the retina. The TA concentration accomplished by IVTs is huge compared to that achieved by TALF. The observed maximum concentration (C_max_) of TA by IVTs in rabbits is 14,434.0 ± 10,768 μg/L [79], whereas that obtained through the topical application of TALF in the same animal model is 252.10 ± 90.00 ng/g [36]. However, higher concentrations are not synonymous with better therapeutic profiles. It is important to emphasize that the therapeutic activity of glucocorticoids is not only determined by their potency and dosage, but also by their bioavailability in the ocular tissue. It is well known that an increment in dose does not necessarily boost effectiveness [80,81]. For instance, in the fluocinolone acetonide (FA) intravitreal implant of 0.2 μg/study day, the C_max_ was barely 1.26 ng/g in the vitreous and 12.2 ng/g in the retina of rabbits [82]; however, the implant proved to be efficient in patients with diabetic macular edema throughout a 36-month follow-up period (improvement ≥15 letters) [80,83]. Low glucocorticoid levels could be of benefit because lower concentrations reduce the risk of toxicity and remain therapeutic [84], as proved by TALF studies [37,38,39].

Finally, different groups have proven that liposomes are suitable vehicles for the delivery of glucocorticoids into the retina when topically applied [36,85]. QuSomes^®^ represents a patented technology of self-forming and stable liposomes that were incorporated into our formulation. As proposed in a previous publication [36], TALF efficiency regarding the delivery of TA into intraocular tissues is related to formulation components other than synthetic PEGylated lipids such as polyethylene glycol (15)-hydroxy stearate or Kolliphor^®^ HS 15. This reagent is a potent, non-ionic solubilizer and emulsifying agent with low toxicity proposed to act as a permeability enhancer that would promote drug transport across cell membranes (increasing the endocytosis rate) and improve drug translocation throughout the paracellular route (it affects actin organization in the cell cytoskeleton with subsequent tight junction opening) [86].

## 5. Conclusions

In conclusion, TALF has the potential to reduce the treatment burden concerning intravitreal anti-VEGF therapy by significantly reducing the number of intravitreal retreatments. It seems that the lower concentration of TA achieved by topical TALF prevents the complications related to the IVTs of this synthetic glucocorticoid, but preserving their therapeutic activity in patients with nAMD. However, using TALF as an adjuvant to anti-VEGF therapy in nAMD should be studied in a larger cohort of patients to efficiently determine its safety and effectiveness.

## Figures and Tables

**Table 1:** Demographic and clinical characteristics of the patients and their study eyes.

	RBZ-PRN	RBZ-TALF	*p*
Age	70.9 ± 10.46	71.5 ± 6.65	0.6795
Gender			0.9577
Male (*n*)	5	3	
Female (*n*)	5	5	
Eye			0.6353
OD (*n*)	5	4	
OS (*n*)	5	4	
Morbidity			0.1675
Hipertension (*n*)	5	2	
Diabetes Mellitus (*n*)	0	3	
Ocular findings			
Pseudophakic (*n*)	2	4	0.2732
Basal BCVA (ETDRS letters)	57.5 ± 13.2	56.1 ± 14.8	0.8113
Basal CFT (μm)	334.5 ± 106.9	399.1 ± 75.6	0.1522

BCVA, best corrected visual acuity; CFT, central foveal thickness; OD, oculus dexter (right eye); OS, oculus sinister (left eye); PRN, pro re nata; RBZ, ranibizumab; TALF, triamcinolone acetonide-loaded liposome formulation.

**Table 2:** Clinical outcomes after TALF administration as an adjuvant to anti-VEGF therapy in nAMD.

	BCVA			CFT			IOP		
	(ETDRS Letters)			(μm)			(mmHg)		
Patient	Baseline	Month 6	Month 12	Baseline	Month 6	Month 12	Baseline	Month 6	Month 12
1	75	75	80	413	334	333	14	14	15
2	60	79	80	316	215	225	13	12	15
3	68	76	*	319	245	*	13	16	*
4	65	65	70	323	286	287	13	14	13
5	39	40	57	452	223	211	14	12	13
6	54	58	65	453	353	182	15	17	14
7	34	35	40	446	272	198	16	16	15
8	72	72	76	493	284	199	12	12	11
9	48	*	*	320	*	*	14	*	*
10	50	72	69	297	217	241	11	14	13
Mean ± SD	56.1 ± 14.8	62.0 ± 16.5	67.1 ± 13.4 †	399.1 ± 75.6	273 ± 52.7 †	234.5 ± 51.4 †	13.5 ± 1.6	13.9 ± 1.9	13.6 ± 1.4

BCVA, best corrected visual acuity; CFT, central foveal thickness; ETDRS, Early Treatment Diabetic Retinopathy Study; IOP, intraocular pressure; SD, standard deviation; TALF, triamcinolone acetonide-loaded liposome formulation; * out of protocol (compliance failure); † statistically significant from baseline.

**Table 3:** Comparison of efficacy endpoints at the end of the follow-up period between the RBZ-PRN and RBZ-TALF groups.

	RBZ-PRN		RBZ-TALF		*p*
	Baseline	Month 12	Baseline	Month 12	between Groups
Average BCVA (ETDRS letters)	57.5 ± 13.2	63 ± 15.49	56.1 ± 14.8	67.1 ± 13.4	0.6484
Gain in ETDRS letters		5.5 ± 14.99		11.0 ± 7.0	0.4445
Average CFT(μm)	334.5 ± 106.9	237.2 ± 31.13	399.1 ± 75.6	234.5 ± 51.4	0.6484
CFT change (μm)	97.3 ± 95.29	97.3 ± 95.29		164.6 ± 108.1	0.1525
Intravitreal injections (*n*)		6.1 ± 1.28		2.5 ± 1.41	0.0004

BCVA, best corrected visual acuity; CFT, central foveal thickness; ETDRS, Early Treatment Diabetic Retinopathy Study; PRN, pro re nata; RBZ, ranibizumab; TALF, triamcinolone acetonide-loaded liposome formulation.

## Data Availability

The data presented in this study are available upon request from the corresponding author. The data are not publicly available due to privacy and ethical restrictions.
